# *HECTD2* Is Associated with Susceptibility to Mouse and Human Prion Disease

**DOI:** 10.1371/journal.pgen.1000383

**Published:** 2009-02-13

**Authors:** Sarah E. Lloyd, Emma G. Maytham, Hirva Pota, Julia Grizenkova, Eleni Molou, James Uphill, Holger Hummerich, Jerome Whitfield, Michael P. Alpers, Simon Mead, John Collinge

**Affiliations:** 1 MRC Prion Unit, University College London Institute of Neurology, London, United Kingdom; 2 Department of Neurodegenerative Diseases, University College London Institute of Neurology, London, United Kingdom; 3 Papua New Guinea Institute of Medical Research, Goroka, Eastern Highlands Province, Papua New Guinea; 4 Centre for International Health, Curtin University, Perth, Australia; McLaughlin Research Institute, United States of America

## Abstract

Prion diseases are fatal transmissible neurodegenerative disorders, which include Scrapie, Bovine Spongiform Encephalopathy (BSE), Creutzfeldt-Jakob Disease (CJD), and kuru. They are characterised by a prolonged clinically silent incubation period, variation in which is determined by many factors, including genetic background. We have used a heterogeneous stock of mice to identify *Hectd2*, an E3 ubiquitin ligase, as a quantitative trait gene for prion disease incubation time in mice. Further, we report an association between *HECTD2* haplotypes and susceptibility to the acquired human prion diseases, vCJD and kuru. We report a genotype-associated differential expression of *Hectd2* mRNA in mouse brains and human lymphocytes and a significant up-regulation of transcript in mice at the terminal stage of prion disease. Although the substrate of HECTD2 is unknown, these data highlight the importance of proteosome-directed protein degradation in neurodegeneration. This is the first demonstration of a mouse quantitative trait gene that also influences susceptibility to human prion diseases. Characterisation of such genes is key to understanding human risk and the molecular basis of incubation periods.

## Author Summary

Prion diseases are fatal transmissible neurodegenerative diseases of animals and humans for which there is no treatment. They include Bovine Spongiform Encephalopathy (BSE), and its human equivalent, variant Creutzfeldt-Jakob Disease (vCJD). Prion diseases are characterised by a long, silent incubation period before the disease emerges, and this time interval varies greatly between individuals. Differences in our genetic makeup are a key factor in this variability. We already know that natural variation within one key gene, the prion protein gene, has a major influence on incubation time, but it is now clear that a number of other genes are also important. Using a mouse model, we have identified one of these genes, *Hectd2*, which is thought to be involved in the process that removes unwanted proteins from the cell. We also show that *HECTD2* is associated with an increased risk of two human prion diseases—vCJD in the United Kingdom and kuru in Papua New Guinea. These data will give us a better understanding of the fundamental processes involved in these diseases and go some way to explaining why some individuals exposed to BSE have developed vCJD and others have not.

## Introduction

Prion diseases are fatal transmissible neurodegenerative disorders of animals and humans. These include the agriculturally and economically important diseases of scrapie and Bovine Spongiform Encephalopathy (BSE) and the human diseases sporadic Creutzfeldt-Jakob disease (CJD), variant (vCJD) and kuru. Sporadic CJD has no known aetiology and vCJD is thought to have arisen following exposure to BSE prions [1]. Kuru is a prion disease that reached epidemic proportions in the 1950s in the Fore linguistic region of Papua New Guinea and is thought to have been transmitted through endocannibalism by participation in mortuary feasts [2]. Following the cessation of this practice in the late 1950's, the incidence of disease has declined, however, it remains our only experience of a large epidemic of acquired human prion disease and provides a useful model for vCJD [3].

Although there was widespread population exposure in the UK and some other countries to BSE only around 200 have developed clinical vCJD to date, although the number infected remains unknown. This represents an on-going public health concern with a risk of iatrogenic transmission through blood and surgical instruments. vCJD has not been associated with any unusual pattern of dietary or occupational exposure to BSE prions and a significant genetic component to risk seems probable therefore the identification of susceptibility factors is key to estimating individual risk [1].

All prion diseases have prolonged clinically silent incubation periods which in humans span over 50 years [2]. Marked variation in incubation period occurs between inbred lines of mice and this is determined by multiple genetic loci in addition to the prion protein gene [4],[5]

Previous studies have identified several quantitative trait loci for prion disease incubation time in mice. However, the resulting regions of interest spanned many megabases and were consequently too large for individual candidate gene analysis [6]–[10]. Several different strategies are available for fine mapping [11]–[13] and we chose to use a heterogeneous stock of mice. These are produced to model an out-bred population of mice, however they have the advantage of starting with a defined number of parental alleles. Heterogeneous stocks of mice have been shown to be a useful mapping tool because they provide a high level of recombination and the development of specific mapping software allows for convenient multipoint linkage analysis [14],[15]. This approach led to the identification of *Hectd2*, an E3 ubiquitin ligase, as a quantitative trait gene for prion disease incubation time. Mouse models are extremely useful for studying human prion diseases as they faithfully recapitulate many key features of the disease and indeed rodents are naturally susceptible to prion diseases. It is expected that susceptibility genes and pathways identified in mice will also be relevant to human prion diseases. To test this hypothesis we carried out an association study with *HECTD2* markers and samples from different human prion diseases and successfully found a significant association with two acquired forms of prion disease: vCJD and kuru.

## Results

### Identification of Mouse Quantitative Trait Gene

To fine map regions thought to contain quantitative trait loci for prion disease incubation time we utilised the Northport heterogeneous stock [16] (HS) of mice (gift of Robert Hitzemann), which was produced by semi-randomly mating eight inbred lines of mice (A/J, AKR/J, BALB/cJ, C3H/HeJ, C57BL/6J, CBA/J, DBA/2J, LP/J). Approximately 1000 mice were inoculated intracerebrally with mouse-adapted scrapie prions (Chandler/RML) and incubation times (in days) were determined as previously described [6],[17]. Regions of interest for fine mapping include those identified in previous crosses [6]–[10] and also from other studies. In this study we focus on a region of Mmu19 as a result of an interest in candidate genes on human chromosome 10 (unpublished data).

Nine microsatellite markers from chromosome 19 (*D19Mit86-D19Mit112* see Table S1) at approximately 1 cM intervals were genotyped in approximately 400 animals which represent the extreme 20% of both sides of the incubation time distribution. Multipoint linkage analysis was carried out using HAPPY (http://www.well.ox.ac.uk/happy) [18]. A peak of linkage (−log*P* = 5.88) was seen between *D19Mit63* and *D19Mit65*, a region of approximately 2.9 Mb (Figure S1A). Significant linkage was taken as −log*P*>3 as defined by a permutation test (n = 1000) carried out by HAPPY. This interval explains 6.9% of the observed variance therefore as predicted by other QTL mapping studies [6]–[10], other loci are expected to contribute to prion disease incubation time. Trait estimates for each strain are shown in Table S2.

Twenty seven RefSeq genes were identified within this region (NCBI build 37), 22 of which were sequenced (Table S3) in the parental strains of the HS in order to identify polymorphisms. Sequencing was not exhaustive and focused primarily on the exons including 5′ and 3′UTRs, intron/exon boundaries and potential promoters as defined by the literature for each gene or PROSCAN (http://www-bimas.cit.nih.gov/molbio/proscan). 177 polymorphisms were identified across the region which included single nucleotide polymorphisms (SNPs), simple repeats and insert/deletion polymorphisms. Most of the variation was observed in non-coding regions, however, several non-synonymous changes were seen (for detail see Table S4). All variants were assessed using an additional function of HAPPY which assigns a probability that any polymorphism is a quantitative trait nucleotide (QTN) [19]. This predicts which strain distribution pattern (SDP) most closely fits the pattern identified by the microsatellites in the HS animals (Figure S1B, Table S4). The main candidates to emerge from this analysis are *Hectd2*, *Exoc6*, *Cyp26c1*, *Cyp26a1*, *Plce1 and Lgi1* (Table 1). Some SDPs are broadly conserved across the whole gene (e.g. −log*P* = 6.12 *Hectd2*, −log*P* = 6.74 *Cyp26c1* and *Plce1*) whereas others represent single polymorphisms (e.g. −logP = 6.74 *Cyp26a1* and *Lgi1*).

**Table 1:** Most significant strain distribution patterns.

Strain distribution pattern	Genes	−logP	Comment
(A, AKR, BALB) (C3H, C57, CBA, DBA, LP)	*Hectd2*	6.12	Promoter, Several intronic and 3′UTR
(A, AKR BALB) (C3H, CBA, DBA, LP) (C57)	*Hectd2*	6.15	Single intronic
(A, AKR, BALB, C57, LP) (C3H, CBA, DBA)	*Exoc6*	6.74	2 intronic, 1 3′UTR
	*Cyp26c1*		T18A, Q256R and other synonymous
	*Cyp26a1*		G202D
	*Plce1*		Several intronic and synonymous
	*Lgi1*		Single intronic
(A, AKR, BALB, C57) (C3H, CBA, DBA, LP)	*Hectd2*	6.84	Single SNP 3′UTR
	*Cyp26a1*		Single intronic

−logP values are estimated by HAPPY based on polymorphisms detected in the parental strains of the HS.

The −log*P* values assigned by HAPPY are predictions. We therefore tested representative polymorphisms from either each gene, or each strain distribution pattern, by genotyping the HS (Table 2). The only highly significant SNPs were seen in *Hectd2* (*P* = 0.0008, 0.0013 and 0.0022, ANOVA) suggesting that *Hectd2* is the most promising candidate in this region. However, these analyses are not exhaustive and it is not possible to exclude the possibility that variation in other genes or intergenic regions also contribute to prion disease incubation time.

**Table 2:** Polymorphism genotyping in HS mice.

Gene	Polymorphism	Happy −logP	HS p-value (ANOVA)
*Hectd2*	Promoter (G)n	6.12	P = 0.0008 (n = 398)
	Intron 3 A/G	6.12	P = 0.0013 (n = 404)
	3′UTR A/T	6.84	P = 0.0022 (n = 359)
*Cyp26c1*	Exon 1 T18A	6.74	P = 0.1512 (n = 403)
*Cyp26a1*	Exon 3 G202D	6.74	P = 0.2017 (n = 408)
*Plce1*	Exon 6 T/C	6.74	P = 0.1556 (n = 411)

All polymorphisms were analysed by allele discrimination using a 7500 Fast real time PCR system (Applied Biosystems) except the *Hectd2* promoter polymorphism which was typed by size using fluorescent primers on a MegaBACE1000 sequencer (GE Healthcare). For probe details see Table S5.

Seven SNPs were identified in the 3′UTR of *Hectd2* (Table S4), however, it is not clear whether they would affect regulation. Five polymorphisms occur within the predicted promoter (−226 to +25) one of which affects a potentially functional site. Sequence for the C57BL6/J allele from −216 to −210 is T**GGGCGG** and the insertion of 6 Gs in the alternative allele gives TGGGGGG**GGGC** **GG** . Both variants contain the consensus sequence for a Sp1 binding site (shown in bold) however the insertion also generates an overlapping large T antigen binding site (underlined). It is unclear whether additional mouse proteins could bind to this sequence or whether Sp1 binding would be affected. The significant SDPs were spread across the whole of *Hectd2* therefore we cannot exclude any of these closely linked polymorphisms either individually or collectively from a contribution to the phenotype.

### Mouse *Hectd2* Expression

**Figure 1:**
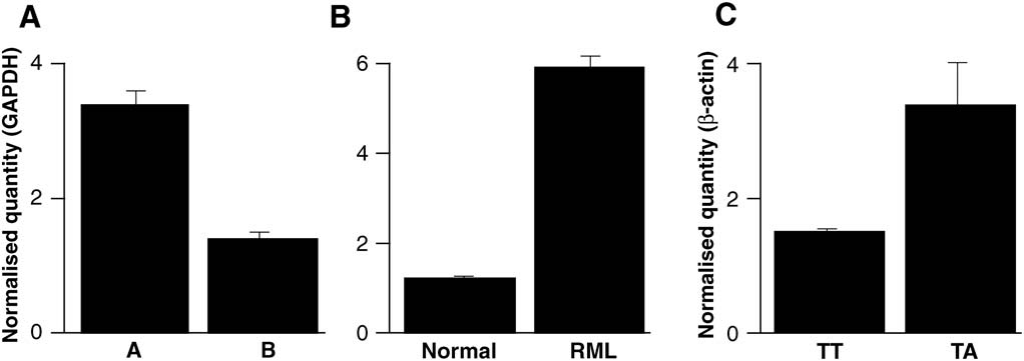
Quantitative RT-PCR of *Hectd2I*. cDNA was prepared from whole brains of uninfected 8 week old male mice or mice at the terminal stages of disease following intracerebral inoculation with Chandler/RML mouse-adapted scrapie prions. All samples were duplexed for *Hectd2* and *GAPDH* fluorogenic probes and run in triplicate with n = 6 for each mouse strain/group. Mean±s.e.m. *Hectd2* mRNA expression level is expressed in arbitrary units as normalised by the quantity of *GAPDH* (*y*-axis). A, Inbred strains are grouped according to the major strain distribution pattern seen in *Hectd2* (Group A = A, AKR, BALB; Group B = C3H, C57, CBA, DBA). Expression was ×2.4 greater in group A than group B (*P* = 2.85×10^−9^, unpaired *t*-test). B, Comparison of *Hectd2* expression in normal and RML prion-infected C57BL6 mouse brains. Expression was ×5.0 greater in the brains of prion-infected mice, (*P* = 2.66×10^−8^, unpaired *t*-test). C, Expression of *HECTD2* in cDNA prepared from lymphocytes of human blood donors (n = 140). Samples were duplexed for *HECTD2* and *β-actin* fluorogenic probes and run four times. Mean±s.e.m. *HECTD2* mRNA expression level is expressed in arbitrary units as normalised by the quantity of β-actin (*y*-axis). Data are grouped according to genotypes at *rs12249854* as determined from genomic DNA. Expression was ×2.3 greater in the heterozygotes (TA) than for the major allele homozygotes (TT) (*P* = 0.0008 Mann-Whitney test).

To determine whether the polymorphisms detected in *Hectd2* have an effect on expression, RNA was extracted from whole brains of 8 week old males from the parental strains of the HS (except LP). Samples were analysed by real time RT-PCR. To examine genotype-related differential expression, strains were grouped according to the major strain distribution pattern seen in *Hectd2* (Group A = A, AKR, BALB; Group B = C3H, C57, CBA, DBA). Expression was ×2.4 greater in group A than group B (*P* = 2.85×10^−9^, unpaired *t*-test) (Figure 1A). Where incubation time data are available, the increase in *Hectd2* expression is associated with a shorter incubation time (R^2^ = 0.61) [20]–[22] (See also Figure S2). A potential role for *Hectd2* in prion disease pathogenesis was explored by comparing the mRNA expression levels between normal mice and those at the end stage of disease following infection with Chandler/RML prions. For C57BL/6, expression was ×5.0 greater in the prion infected mice (*P* = 2.66×10^−8^, unpaired *t*-test) (Figure 1B).

### Human Association Study

Our data indicate that *Hectd2* influences prion disease incubation time in mice. We therefore analysed *HECTD2* in a hypothesis-driven association study of human prion disease. We analysed 834 samples from patients with prion disease or strong resistance to prion disease and 1162 relevant control population samples. We tested whether genetic variation at *HECTD2* was associated with a phenotype of variant and sporadic CJD. In Papua New Guinea (PNG) we genotyped patients who died from the epidemic prion disease kuru, transmitted by endocannibalism, and compared these data with elderly women known to have had multiple exposures to kuru at mortuary feasts prior to the cessation of endocannibalism in the late 1950's, but who are long-term survivors [2],[23]. See methods for details of the patient data, populations, phenotype ascertainment and population stratification data.

We initially tested a single SNP, *rs12249854*(A/T), located in a *HECTD2* intron, and showed that the minor allele (A) was significantly over-represented in vCJD (n = 117, 8.1%) compared to controls (n = 601, 3.9%), *P* = 0.0049, (OR 2.11, 95% CI 1.19–3.77, trend test 1 d.f.), and between sporadic CJD (n = 452, 6.3%) and controls, *P* = 0.012, (OR 1.65, 95% CI 1.11–2.46, trend test 1 d.f.). Given that sample sizes are necessarily small in both sporadic and variant CJD, these data are consistent with the association of *rs12249854* with risk in both prion disease categories and a large effect size. We went on to test whether the risk *rs12249854* allele modified the phenotype of human prion disease. Although the age of onset of sporadic CJD with *rs12249854*AA was younger than other genotypes (53.5 years Vs 68.8 years for *rs12249854*AT and 69.0 years for *rs12249854*TT, *P* = 0.048 *t*-test), this genotype was rare (n = 3) and the finding therefore was not robust. There was no association between vCJD year of presentation, or age of onset with *rs12249854* genotype. Insufficient data were available to look at any association with duration of illness.

We went on to analyse a further seven SNPs in *HECTD2* selected to capture global genetic diversity based on Hapmap (http://hapmap.org) [24] data (Table S6). In the United Kingdom (UK), we found strong linkage disequilibrium (LD) and a simple haplotype structure across the entire gene (Table 3). Three haplotypes were >1% frequency, the most common two haplotypes (1 and 2) differed at all SNPs, a third haplotype (3) was distinguished from the most common haplotype (1) by a single SNP upstream of *HECTD2*. Increased risk of vCJD was associated with haplotype 2, possessing *rs12249854*A, but the extensive LD prevented us from identifying the functional SNP. In PNG, however, we found considerably more diversity with four common haplotypes, 1, 2 and two novel haplotypes 4 and 5 (see methods for haplotype inference). Haplotype 2, most significantly associated with vCJD (haplotype association test, *P* = 0.006), showed no significance between kuru and the elderly female survivors of mortuary feasts. Rather, in PNG we found that a population specific haplotype (designated 4) was strongly associated with kuru (*P* = 0.0009). Haplotype 4 differs from haplotype 2 at a single SNP, *rs12247672*, which itself is significant in vCJD (*P* = 0.0039) but not at all in kuru (P = 0.6138). Our data suggest that there is evidence for HECTD2 association in both vCJD and kuru however the functional polymorphisms are likely to be different. This is not necessarily surprising given the distinct evolutionary history and consequent genetic differences that exist between the UK and PNG populations. It should also be noted that although vCJD and kuru are both acquired human prion diseases that share many characteristics they are also derived from different sources and caused by distinct prion strains [25],[26] therefore the mechanism of HECTD2 involvement may also be different.

**Table 3:** Most common HECTD2 haplotype frequencies.

UK				
Haplotype	Name	vCJD	Control	P-value
22222222	1	0.951	0.912	0.02
**11111111**	**2**	**0.026**	**0.061**	**0.006**
12222222	3	0.023	0.027	0.73

Papua New Guinea				
Haplotype	Name	Multiple exposure	Kuru	P-value
22222222	1	0.641	0.743	0.014
11111111	2	0.146	0.123	0.45
**12111111**	**4**	**0.173**	**0.076**	**9×10^−4^**
22222211	5	0.040	0.058	0.36

1 = minor allele; 2 = major allele.

### *HECTD2* Sequencing

We sequenced the ORF and promoter of *HECTD2* in 16 vCJD, multi kuru-exposure survivors, and both UK and PNG controls. Three polymorphisms were found, of which only one is potentially functional (Table 4). *rs7081363* occurs in the promoter (−247) and the minor allele is predicted to remove an Sp1 binding site (GGC**G/A**GG). *rs7081363* was genotyped in our samples and shown to be in complete LD with *rs12249854* in the UK population (vCJD *P* = 0.0012; sporadic CJD *P* = 0.0065). We were unable to genotype the kuru samples due to poor DNA quality, however, analysis of all other samples suggest that *rs7081363* is unlikely to be significant in PNG.

**Table 4:** HECTD2 polymorphisms.

Location	ID	UK Genotyping	PNG Genotyping
Promoter (−247)	*rs7081363* (G/A)	vCJD (n = 114), P = 0.0012	Multiexposure (n = 93)
		sCJD (n = 425), P = 0.0065	Unexposed Fore (n = 128), P = 0.9969
		Controls (n = 616)	Unexposed PNG (n = 275), P = 0.2836
			Kuru - ND
Promoter (−184)	C/A	ND	ND
Exon 4	*rs7920604* (G/A)	ND	ND

Sequencing of the open reading frame and putative promoter was carried out in 16 vCJD samples, 16 multiple exposure unaffected elderly women from PNG, 16 UK controls and 16 controls from PNG.

*rs7081363* was genotyped by allelic discrimination on a Real Time PCR machine (Applied Biosystems). It was not possible to genotype the kuru sample due to the quality of the DNA. The minor allele is in linkage disequilibrium (LD) with *rs12249854* in the UK but not in PNG. The minor allele (A) eliminates an Sp1 binding site on the negative strand (−250 to −245).

Based on the sequencing results the minor allele at promoter polymorphism at −184 and *rs7920604* appear to be in LD with *rs12249854* in both the UK and PNG.

PNG – Papua New Guinea, ND – not done.

### Human *HECTD2* Expression

To determine whether the susceptibility alleles in the UK population are associated with differential mRNA expression, *HECTD2* expression levels in blood lymphocytes (n = 140, UK blood donors) were quantified by real-time RT-PCR. Samples were grouped according to *rs12249854* genotype, however, due to the low frequency of the minor allele (A), no homozygotes (AA) were seen. The mean expression level was ×2.3 greater in the heterozygotes than for the major allele homozygotes (TT) (*P* = 0.0008 Mann-Whitney test, Figure 1C). This suggests that a higher level of *HECTD2* mRNA expression may be linked with vCJD in the UK population.

## Discussion

Our data show that *HECTD2* is linked to prion disease incubation time in mouse and is associated with sporadic and variant CJD and kuru in humans and an increase in expression is associated with a susceptibility genotype and disease pathogenesis. In mouse, we cannot exclude the possibility of other nearby genes or intergenic regions also being implicated as our sequencing studies were not exhaustive. However, in human, the LD block, based on HapMap [24] data, includes only *HECTD2* and does not extend into the neighbouring genes suggesting that the association observed stems from *HECTD2* and not any other gene in the area.

In mouse, the promoter, 3′UTR polymorphisms and the associated differential expression suggest a mechanism by which *Hectd2* may influence the incubation time phenotype. Similarly, in the UK population a promoter polymorphism is also associated with a susceptibility phenotype and a resulting increase in expression level. This suggests that the mode of HECTD2 action in prion disease may be independent of host and prion strain. Due to lack of available material it has not been possible to replicate these experiments in our kuru samples, however, our haplotype study suggest that a different polymorphism is likely to be functional in the PNG population. This does not rule out the possibility that differential expression is also important in PNG, through an alternative polymorphism, although this may be difficult to determine. Our expression analysis in terminally sick mice suggest that HECTD2 is upregulated during the course of infection therefore we can speculate that a higher base line of expression reduces the time taken to reach a threshold level thereby reducing the incubation time.

The ubiquitin-proteosome system has been implicated in the pathogenesis of several neurodegenerative diseases which show an accumulation of an abnormally folded protein including prion disease, Parkinson's disease and Alzheimer's disease [27]–[29]. By homology to other family members, HECTD2 is an E3 ubiquitin ligase suggesting that common pathways are involved in the neurodegenerative processes of these different diseases. Specifically, the mouse *mahoganoid* coat-colour mutation is found in the gene *Mahogunin* which is an E3 ubiquitin ligase [30]. A null mutation of *Mahogunin* causes an age-related progressive neurodegenerative phenotype characterised by spongiform degeneration, neuronal loss and astrocytosis. The phenotype resembles that of prion disease however there is no PrP^Sc^ accumulation. Mutations in the E3 ubiquitin ligase parkin are associated with autosomal recessive juvenile parkinsonism and loss of ubiquitin-protein ligase activity in patients has been shown to be associated with protein accumulation [31]. E3 ubiquitin ligases have also been implicated in the pathogenesis of polyglutamine diseases in particular it has been shown that mutations in the E6-AP ubiquitin ligase reduces the frequency of nuclear inclusions in mice expressing mutant ataxin-1 while accelerating the Purkinje cell pathology [32]. Further, *HECTD2* maps to a region of human chromosome 10q previously linked with Alzheimer's disease [33] suggesting that *HECTD2* may also be a susceptibility factor for Alzheimer's disease and other neurodegenerative disorders.

Group sizes for vCJD, kuru and elderly female survivors of mortuary feasts are of necessity small, however we believe that the combined weight of data from the mouse genetic studies, expression analyses and our association study of independent human prion diseases from different populations provide sufficient evidence to support a role for HECTD2 in prion disease. This supports a significant role for the ubiquitin-proteasome system in prion pathogenesis [27],[34],[35] and will contribute to modelling and understanding genetic risk of developing prion disease following BSE and secondary human prion exposure.

## Materials and Methods

### Human Samples

The clinical and laboratory studies were approved by the local research ethics committee of University College London Institute of Neurology and National Hospital for Neurology and Neurosurgery and by the Medical Research Advisory Committee of the Government of PNG. Full participation of the PNG communities involved was established and maintained through discussions with village leaders, communities, families and individuals.

#### vCJD

118 probable or definite vCJD patients, according to established criteria (http://www.advisorybodies.doh.gov.uk/acdp/tseguidance/tseguidance_annexb.pdf), were recruited by the National Prion Clinic (NPC), London or the National CJD Surveillance Unit (NCJDSU), Edinburgh from 1995 to 2005. Iatrogenic vCJD, acquired through blood transfusion was not included in this panel. Genomic DNA was usually extracted from peripheral blood; brain tissue was used as a source for some patients. Amplified DNA, using either multiple displacement amplification (Geneservice, Cambridge, UK) or fragmentation-PCR methods (Genomeplex, Sigma), was used for a small number <10% of samples. Samples were checked for degradation on 1% agarose gel and stored at 50 ng/µl in low concentration Tris-EDTA buffer. All patients were thought to have acquired the disease in the UK and were of white-British ethnicity; 60% were male. Mean (range) age of onset of disease onset was 29 years (13–62).

#### Sporadic CJD

458 probable or definite sporadic CJD patients, according to WHO criteria, were recruited by the National Prion Clinic (NPC), London or the National CJD Surveillance Unit (NCJDSU), Edinburgh, or numerous other referrers in the UK. DNA was sourced and amplified as for vCJD. All patients were of UK or northern European origin. Although the vast majority of patients were of white-British ethnicity, and all patients of known non-white ethnicity were excluded, this information was based on name and geography for some samples. DNA preparation and storage was similar to vCJD. Over 60% had pathologically confirmed sCJD, the remainder had a diagnosis of probable sCJD according to published WHO criteria with a high specificity [36]. Mean (range) age of onset of disease was 62 years (15–87).

#### Kuru/elderly women resistant to kuru

Prior to 1987, kuru surveillance was conducted by many different investigators (Gajdusek, Zigas, Baker, Alpers, Hornabrook, Moir and others) and from 1987 to 1995 solely by the Kuru Surveillance Team of the Papua New Guinea Institute of Medical Research. From 1996 onwards, kuru surveillance was strengthened and a field base and basic laboratory for sample processing and storage was established in the village of Waisa in the South Fore [37]. The kuru collection (n = 151) comprises young children, adolescents and adults from around the peak of the epidemic and elderly recent kuru cases with long incubation times. They resided in the South Fore (53), North Fore (40), Gimi (3), Keiagana (10), or other linguistic groups (11) of the kuru-affected region of the Eastern Highlands Province of Papua New Guinea; in 34 cases the linguistic group within the region was not recorded.

Elderly exposed women were defined as aged over 50 years in 2000 from a kuru-exposed region (n = 115). These women were unaffected at the time of sampling but were thought to have been exposed to kuru prions in childhood. Although these women may not be truly “resistant” to kuru prions they would have incubation times in excess of 40 years. Additional controls were obtained from the young modern day healthy population that has not been exposed to kuru but came from villages in the exposed region by matching each elderly woman (“resistant”) to at least two current residents of the same village aged less than 50 in 2000. These largely came from the South Fore, but with a significant number from the North Fore and a small number of individuals from Gimi, Keiagana and Yagaria linguistic groups. Further controls were obtained from young unexposed people from areas of PNG where no kuru has been recorded. Where identified by either genealogical data or microsatellite analysis, first degree relatives were excluded from these groups. DNA from degraded archival kuru sera, obtained from the NIH collection, was isolated by QIAGEN QIAamp Blood DNA minikit followed by whole genome amplification either through using a Φ29 protocol (Geneservice), or GenomePlex Complete Whole Genome Amplification Kit (WGA2) (Sigma).

#### UK controls

116 individuals were recruited from the National Blood Service (NBS). Information was collected about gender, age, ethnicity and birthplace divided into 12 regions. Samples were very similar to vCJD for white-British ethnicity, birthplace (by 12 regions in UK) and gender. DNA was extracted from whole blood. PAXgene blood RNA samples were also collected (Preanalytix). Mean (range) of age at sampling was 34 years (18–64); 56% were male. Further UK control samples (n = 480) were purchased from the European Collection of Cell Cultures (ECACC) Human Random control (HRC) DNA panels consisting of randomly selected, non-related UK Caucasian blood donors. Total number of UK controls was n = 596.

#### Population structure

Population structure was considered with identity by state (IBS) clustering (implemented by PLINK (http://pngu.mgh.harvard.edu/˜purcell/plink/index.shtml), and principal components analysis (implemented by the EIGENSTRAT package [38]. Genome-wide data (manuscript in preparation) with high stringency filtering was used to compare vCJD and UK controls with both PLINK or EIGENSTRAT (no significant eigenvectors were detected using default procedures). For other groups of patients and PNG groups genotypes were generated for 1325 SNPs in 344 randomly selected, non-related UK Caucasian blood donors provided by the European Collection of Cell Cultures (see above, methods), 458 sCJD patients, 143 kuru patients, 115 elderly women resistant to kuru born before 1950, and 282 young individuals from the kuru region matched to the village of residence of the elderly women. We used the Illumina Goldengate platform at the St. Bartholomew's Hospital Genome Centre. SNPs were filtered for association with vCJD by comparison with UK controls by best permuted P<0.001 from any of 4 genetic models (allelic, trend, genotypic or recessive). Genotyping quality was assessed by Hardy-Weinberg equilibrium (excluding those by exact test *P*<0.001) and visual inspection of all genotype clusters with Beadstudio v3.1. Overall genotype call rate was 99.7%, concordance of duplicate samples was >99.7%. All autosomes were equally represented with a median intermarker distance of 1.3 MB. For EIGENSTRAT 10 eigenvectors were generated using default procedures and outlier detection (6 PNG samples were removed). No significant eigenvectors (*P*>0.01) were identified between sCJD, iatrogenic CJD and UK controls, or between kuru patients, elderly women resistant to kuru and healthy young Fore (total 5 comparisons).

#### Statistical analysis of human data

PLINK was used for association and permutation testing. The primary analysis was a trend model chi-squared test. Haplotype inference was made with PLINK and Haploview using standard expectation-maximisation algorithms. The haplotype association test was implemented through PLINK.

### Animals

28 pairs of Northport HS mice were obtained from R. Hitzemann (Portland, Oregon, USA) at generation 35. Offspring from these pairs were randomly mated to produce a total of 49 pairs. 1000 offspring (generation 37) were used for inoculation. All other inbred lines were obtained from Harlan, UK. Mice were identified by individual transponder tags (Trovan) and tail biopsies were obtained for DNA extraction. Mice were anaesthetized with isofluorane/O_2_ and inoculated intra-cerebrally into the right parietal lobe with 30 µl Chandler/RML prions as previously described [6]. Incubation time was calculated retrospectively after a definite diagnosis of scrapie had been made and defined as the number of days from inoculation to the onset of clinical signs [17]. All procedures were conducted in accordance with UK regulations (Local ethics approval and Home Office regulation) and international standards on animal welfare.

#### Genotyping

Microsatellites were selected (Table S1) from the UCSC Mouse Genome Browser http://genome.ucsc.edu and Mouse Genome Informatics web site (www.informatics.jax.org). For the HS cross nine microsatellite markers from chromosome 19 (*D19Mit86-D19Mit112* see Table S1) were genotyped in approximately 400 animals which represent the extreme 20% of both sides of the incubation time distribution. Fluorescently labelled and standard oligonucleotides were synthesized by Sigma-Genosys. PCR reactions were all carried out in 5 µl on 96-well plates using MegaMix Blue (Microzone Ltd) according to the manufacturer's instructions using 5pmoles of each primer. PCR conditions were determined empirically but in general cycling conditions using a PTC-225 (MJ Research) thermal cycler were as follows: 94°C for 10 min; 94°C 30 s, 55°C 30 s, 72°C 30 s for 35 cycles; 72°C for 5 min. Products of appropriate size and fluorochrome were pooled before further processing. Reactions were ethanol precipitated, washed in 70% ethanol and re-suspended in a total of 10 µl including 5.8 µl MegaBACE loading solution (GE Healthcare) and 0.2 µl MegaBACE ET400-R size standard (GE Healthcare). 1/10 dilution in MegaBACE loading solution was used for analysis. Fragments were heat denatured at 94°C for 2 min before loading onto a MegaBACE1000 capillary sequencer (GE Healthcare). Samples were injected at 3 KV for 45 s and run at 10 KV for 60 minutes. Fragment sizes were analysed using Genetic Profiler v1.1 (GE Healthcare). Multipoint linkage analysis was carried out using HAPPY. Mouse family structure was not taken into consideration for this analysis, therefore, the effect size calculated by HAPPY (http://www.well.ox.ac.uk/happy) [18] for each strain (Table S2) may be overestimated. Novel methods are under development for including family structure and more accurate estimates of effect size (personal communication Richard Mott).

#### Sequencing

Genomic DNA for the parental strains were obtained from the Jackson Laboratory (Bar Harbor, Maine, USA) to minimize any differences with the HS mice due to sub strain variations. PCR products were designed to cover the open reading frame, 5′ and 3′ untranslated region, intron-exon boundaries and potential promoter sequences as defined by the literature for each gene or as predicted by PROSCAN. PCR products were generated in 25 µl reactions using MegaMix Blue (Microzone Ltd) according to the manufacturer's instructions with 10pmole of each primer. Cycling conditions were determined empirically but in general were 94°C for 10 min; 94°C 30 s, 60°C 45 s, 72°C 60 s for 40 cycles; 72°C for 5 min. PCR products were cleaned using Microclean (Microzone Ltd) according to the manufacturer's instructions and re-suspended in H_2_O. 100–200 ng PCR product was added to a 15 µl sequencing reaction including 5pmoles of either the forward or reverse primer, 1 µl BigDye Terminator v1.1 Cycle Sequencing Kit (Applied Biosystems) and 5 µl Better Buffer (Microzone Ltd). Cycling conditions were: 95° 30 s, 50° 15 s, 60° 120 s, for 30 cycles. Reactions were ethanol precipitated, washed in 70% ethanol and re-suspended in 10 µl MegaBACE loading solution (GE Healthcare). Products were detected on a MegaBACE1000 capillary sequencer (GE Healthcare). Samples were injected at 3 KV for 40 s and run at 9 KV for 100 minutes.

### RNA Extraction and Quantitative RT-PCR

#### Mouse brain

RNA was extracted from whole brains from either uninfected or RML terminally sick mice. For the HS parental lines samples were obtained for A, AKR, BALB/c C3H, C57BL/6, CBA and DBA/2 strains but not LP. Eight week old adult male mice were used for all normal brains. Tissue was homogenized using a Ribolyser according to the manufacturer's instructions. RNA was prepared using either RNeasy Maxi (Qiagen) kit or TRIreagent (Ambion) according to the manufacturer's instructions. Samples were treated with DNaseI (Qiagen) and purified further using RNeasy Mini (Qiagen) columns according to the manufacturer's instructions. 4 µg total RNA was reversed transcribed with AMV reverse transcriptase and random primers from the Reverse Transcription System (Promega) according to the manufacturer's instructions. Reactions with no reverse-transcription were also carried out for each sample to ensure no genomic DNA contamination. *Hectd2* real-time PCR was carried out on a 7500 Fast Real-time PCR System (Applied Biosystems) in a total volume of 15 µl using 1 µl cDNA (200–300 ng) and QuantiTect probe PCR kit (Qiagen) according to the manufacturer's instructions. Primers (6 pmoles) F- 5′-CCCCTGAGCTAGGCATTTCC-3′, R-5′ –GAGTTACTGCACCCTTGAATTCTG-3′ and probe (3 pmoles) 5′-FamTGTCGCTGTGCTTTCTGCACCCAACTamra-3′ were designed using PrimerExpress software (Applied Biosystems) and supplied by Sigma Genosys. Rodent GAPDH or β-actin (data not shown) (Applied Biosystems) was duplexed within the reaction as an endogenous control according to the manufacturer's instructions. All reactions were carried out in triplicate using the following cycling conditions: 95°C 15 mins; 95°C 15 s, 60°C 60 s for 40 cycles.

#### Human blood

2.5 ml of blood was collected into PAXgene Blood RNA tubes (PreAnalytix) and RNA was extracted using the PAXgene 96 Blood RNA kit (PreAnalytix) according to the manufacturer's instructions.

1–2 µg of RNA was used to synthesize cDNA in a 20 µl reaction using Omniscript Reverse Transcriptase (Qiagen), 0.5 µg random hexamers (Promega) and 40 u RNasin Plus (Promega). Samples were incubated at room temperature for 10 min followed by 1 hour at 37°C and 65°C for 5 mins. cDNA was diluted 1/2 in H_2_0 for use in downstream PCR reactions. For real-time reactions human *HECTD2* primers (6 pmoles) F- 5′-GCAATGTTACCGTGGACGACTT -3′, R-5′ – CTTCAACATTGCCTTCATGTGATAA -3′ and probe (3 pmoles) 5′-Fam CAAATTATGCCTGAGTTGGCCCATGGAT Tamra-3′ were designed using PrimerExpress software (Applied Biosystems) and supplied by Sigma Genosys. Reactions were carried out on a 7500 Fast Real-time PCR System (Applied Biosystems) in a total volume of 15 µl using 1 µl cDNA and ROXMegaMix Gold (Microzone Ltd) according to the manufacturer's instructions. Human β-actin or PGK-1 (Applied Biosystems) (data not shown) was duplexed within the reaction as an endogenous control according to the manufacturer's instructions. Four replicates were carried out for all samples using the following cycling conditions: 95°C 5 mins; 95°C 15 s, 60°C 60 s for 45 cycles.

For both mouse and human samples, standard curves were constructed for both target and endogenous controls and used to calculate the quantities of both transcripts. *Hectd2* values were normalized by dividing with the quantity of endogenous control.

#### SNP genotyping

Primers and probes for mouse genotyping (Table S5) were designed using criteria defined by Applied Biosystems and PrimerExpress software (Applied Biosystems). MGB probes labelled with either Vic or Fam were purchased from Applied Biosystems and primers for amplification were obtained from Sigma-Genosys. For human SNPs pre-designed allelic discrimination assays were purchased from Applied Biosystems and used according to the manufacturer's instructions. 2.5pmole of each primer and 1pmole of each probe was used for mouse genotyping. All reactions were carried out in 5 µl on a 7500 Fast Real-time PCR System (Applied Biosystems) using either RoxMegaMix Gold (Microzone Ltd) or QuantiTect probe PCR kit (Qiagen). Cycling conditions were the same for both enzymes (95°C 15 s, 60°C 60 s for 40 cycles) however RoxMegaMix Gold and QuantiTect enzyme required 5 and 15 mins initial heating at 95°C respectively. For marker *rs7081363* primers were designed as above. F primer (CCCGACCCGCGACG), R primer (CCCACAGGTCCCACAGGTTT), Probe allele C (CCTCCCCCGCCC – Vic/MGB), Probe allele T (CCTCCCCTGCCC – Fam/MGB). 10 µl reactions were carried out as above except that 1 M Betaine (Sigma) was added to the reaction and the annealing temperature was 58°C.

## Supporting Information

Figure S1 HAPPY multipoint linkage analysis for Mmu19. Results are displayed on the *y*-axis as −log of the *P* value with cM or Mb distance along Mmu19 on the *x*-axis. A, Log probability plot (additive model) for microsatellites between *D19Mit86* and *D19Mit112*. The peak of linkage is seen for the interval *D19Mit63-D19Mit65*. For details of intervals see Table S1. B, Linkage analysis for all polymorphisms detected in genes in the interval *D19Mit63-D19Mit65*. Details for individual SNPs are given in Table S4.
(0.06 MB DOC)

Figure S2 Quantitative RT-PCR of *Hectd2* for individual mouse strains. cDNA was prepared from whole brains of uninfected 8 week old male mice or mice at the terminal stages of disease following intracerebral inoculation with Chandler/RML mouse-adapted scrapie prions. All samples were duplexed for *Hectd2* and *GAPDH* fluorogenic probes and run in triplicate with n = 6 for each mouse strain/group. Mean±s.e.m. *Hectd2* mRNA expression level is expressed in arbitrary units as normalised by the quantity of *GAPDH* (*y*-axis). All mouse strains carry the *Prnp^a^* allele. Published incubation times with Chandler/RML are: AKR 123±4; BALB/c 124±11; C3H 132±4; DBA/2 134±3, C57BL/6 137±0 or 143±4; CBA 140±10 [20]–[22]. Incubation times with RML prions are unknown for mouse strains A and LP.
(0.02 MB DOC)

Table S1 Linkage analysis for microsatellite markers Mmu19.
(0.03 MB DOC)

Table S2 Trait estimates for HS parental strains *D19Mit63-D19Mit65*.
(0.03 MB DOC)

Table S3 Sequenced RefSeq genes from interval *D19Mit63-D19Mit65*.
(0.03 MB DOC)

Table S4 Analysis of polymorphisms from *D19Mit63-D19Mit65*.
(0.3 MB DOC)

Table S5 Primer and probe sequences for mouse polymorphism genotyping.
(0.04 MB DOC)

Table S6 Genotyping results for HECTD2 tagging SNPs.
(0.04 MB DOC)

## References

[1] Collinge J (1999). Variant Creutzfeldt-Jakob disease.. Lancet.

[2] Collinge J, Whitfield J, McKintosh E, Beck J, Mead S (2006). Kuru in the 21st century–an acquired human prion disease with very long incubation periods.. Lancet.

[3] Collinge J, Whitfield J, McKintosh E, Frosh A, Mead S (2008). A clinical study of kuru patients with long incubation periods at the end of the epidemic in Papua New Guinea.. Philos Trans R Soc Lond B Biol Sci.

[4] Carlson GA, Westaway D, Prusiner SB, Prusiner SB, Collinge J, Powell J, Anderton B (1992). The Genetics of Prion Susceptibility in the Mouse.. Prion Diseases in Humans and Animals.

[5] Lloyd S, Collinge J (2005). Genetic Susceptibility to Prion Diseases in Humans and Mice.. Current Genomics.

[6] Lloyd S, Onwuazor ON, Beck J, Mallinson G, Farrall M (2001). Identification of multiple quantitative trait loci linked to prion disease incubation period in mice.. Proc Natl Acad Sci USA.

[7] Lloyd S, Uphill JB, Targonski PV, Fisher E, Collinge J (2002). Identification of genetic loci affecting mouse-adapted bovine spongiform encephalopathy incubation time in mice.. Neurogenetics.

[8] Stephenson DA, Chiotti K, Ebeling C, Groth D, DeArmond SJ (2000). Quantitative trait loci affecting prion incubation time in mice.. Genomics.

[9] Manolakou K, Beaton J, McConnell I, Farquar C, Manson J (2001). Genetic and environmental factors modify bovine spongiform encephalopathy incubation period in mice.. Proc Natl Acad Sci U S A.

[10] Moreno CR, Lantier F, Lantier I, Sarradin P, Elsen JM (2003). Detection of new quantitative trait loci for susceptibility to transmissible spongiform encephalopathies in mice.. Genetics.

[11] Abiola O, Angel JM, Avner P, Bachmanov AA, Belknap JK (2003). The nature and identification of quantitative trait loci: a community's view.. Nat Rev Genet.

[12] Darvasi A (2005). Dissecting complex traits: the geneticists' - 'Around the world in 80 days'.. Trends Genet.

[13] Flint J, Valdar W, Shifman S, Mott R (2005). Strategies for mapping and cloning quantitative trait genes in rodents.. Nat Rev Genet.

[14] Valdar W, Solberg LC, Gauguier D, Burnett S, Klenerman P (2006). Genome-wide genetic association of complex traits in heterogeneous stock mice.. Nat Genet.

[15] Talbot CJ, Nicod A, Cherny SS, Fulker DW, Collins AC (1999). High-resolution mapping of quantitative trait loci in outbred mice.. Nat Genet.

[16] Hitzemann B, Dains K, Kanes S, Hitzemann R (1994). Further-Studies on the Relationship Between Dopamine Cell-Density and Haloperidol-Induced Catalepsy.. J Pharm Exp Therap.

[17] Carlson GA, Kingsbury DT, Goodman PA, Coleman S, Marshall ST (1986). Linkage of prion protein and scrapie incubation time genes.. Cell.

[18] Mott R, Talbot CJ, Turri MG, Collins AC, Flint J (2000). A method for fine mapping quantitative trait loci in outbred animal stocks.. Proc Natl Acad Sci U S A.

[19] Yalcin B, Flint J, Mott R (2005). Using progenitor strain information to identify quantitative trait nucleotides in outbred mice.. Genetics.

[20] Kingsbury DT, Kasper KC, Stites DP, Watson JD, Hogan RN (1983). Genetic control of scrapie and Creutzfeldt-Jakob disease in mice.. J Immunol.

[21] Westaway D, Goodman PA, Mirenda CA, McKinley MP, Carlson GA (1987). Distinct prion proteins in short and long scrapie incubation period mice.. Cell.

[22] Carlson GA, DeArmond SJ, Torchia M, Westaway D, Prusiner SB (1994). Genetics of prion diseases and prion diversity in mice.. Philos Trans R Soc Lond [Biol ].

[23] Mead S, Stumpf MP, Whitfield J, Beck J, Poulter M (2003). Balancing selection at the prion protein gene consistent with prehistoric kuru-like epidemics.. Science.

[24] The International HapMap Project (2003). Nature.

[25] Wadsworth JD, Joiner S, Linehan JM, Asante EA, Brandner S (2008). Review. The origin of the prion agent of kuru: molecular and biological strain typing.. Philos Trans R Soc Lond B Biol Sci.

[26] Wadsworth JD, Joiner S, Linehan JM, Desbruslais M, Fox K (2008). Kuru prions and sporadic Creutzfeldt-Jakob disease prions have equivalent transmission properties in transgenic and wild-type mice.. Proc Natl Acad Sci U S A.

[27] Ciechanover A, Brundin P (2003). The ubiquitin proteasome system in neurodegenerative diseases. Sometimes the chicken, sometimes the egg.. Neuron.

[28] Lim KL (2007). Ubiquitin-proteasome system dysfunction in Parkinson's disease: current evidence and controversies.. Expert Rev Proeomics.

[29] Hegde AN, Upadhya SC (2007). The ubiquitin-proteasome pathway in health and disease of the nervous system.. Trends Neurosci.

[30] He L, Lu XY, Jolly AF, Eldridge AG, Watson SJ (2003). Spongiform degeneration in mahoganoid mutant mice.. Science.

[31] Shimura H, Hattori N, Kubo S, Mizuno Y, Asakawa S (2000). Familial Parkinson disease gene product, parkin, is a ubiquitin-protein ligase.. Nat Genet.

[32] Cummings CJ, Reinstein E, Sun YL, Antalffy B, Jiang YH (1999). Mutation of the E6-AP ubiquitin ligase reduces nuclear inclusion frequency while accelerating polyglutamine-induced pathology in SCA1 mice.. Neuron.

[33] Bertram L, Blacker D, Mullin K, Keeney D, Jones J (2000). Evidence for genetic linkage of Alzheimer's disease to chromosome 10q.. Science.

[34] Kristiansen M, Messenger MJ, Klohn P, Brandner S, Wadsworth JD (2005). Disease-related prion protein forms aggresomes in neuronal cells leading to caspase-activation and apoptosis.. J Biol Chem.

[35] Goldberg AL (2007). On prions, proteasomes, and mad cows.. N Engl J Med.

[36] Poser S, Mollenhauer B, Krauss A, Zerr I, Steinhoff BJ (1999). How to improve the clinical diagnosis of Creutzfeldt-Jakob disease.. Brain.

[37] Collinge J (2008). Review. Lessons of kuru research: background to recent studies with some personal reflections.. Philos Trans R Soc Lond B Biol Sci.

[38] Price AL, Patterson NJ, Plenge RM, Weinblatt ME, Shadick NA (2006). Principal components analysis corrects for stratification in genome-wide association studies.. Nat Genet.

