# Supplementary figures and images for: HECTD2 Is Associated with Susceptibility to Mouse and Human Prion Disease
Source: PLoS Genet. 2009 Feb 13;5(2):e1000383. doi: 10.1371/journal.pgen.1000383 (PMC2633041; doi:10.1371/journal.pgen.1000383)

**Supplementary Information Figure 1**


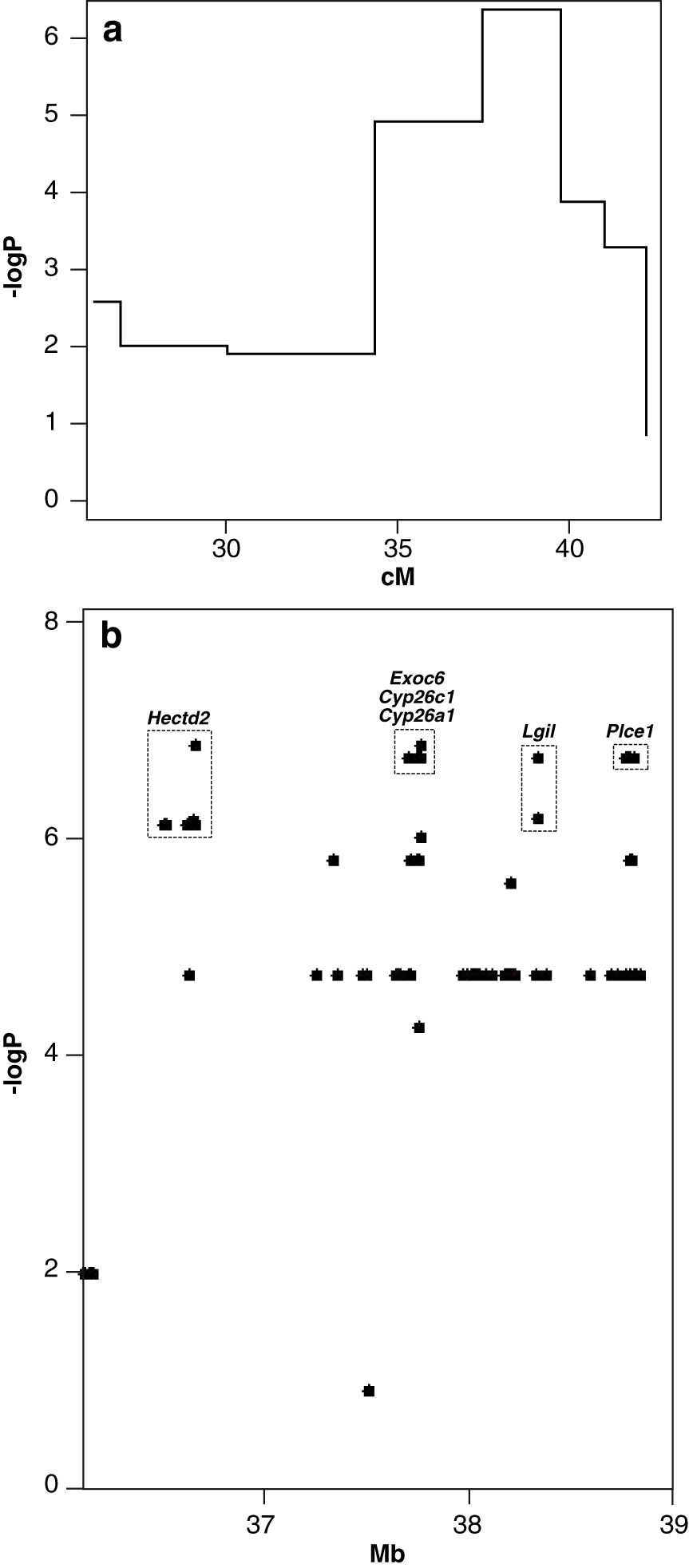

Supplement: Figure S1 — HAPPY multipoint linkage analysis for Mmu19. Results are displayed on the y-axis as −log of the P value with cM or Mb distance along Mmu19 on the x-axis. A, Log probability plot (additive model) for microsatellites between D19Mit86 and D19Mit112. The peak of linkage is seen for the interval D19Mit63-D19Mit65. For details of intervals see Table S1. B, Linkage analysis for all polymorphisms detected in genes in the interval D19Mit63-D19Mit65. Details for individual SNPs are given in Table S4. (0.06 MB DOC) [file pgen.1000383.s001.doc]

**Supporting Information Figure 2**


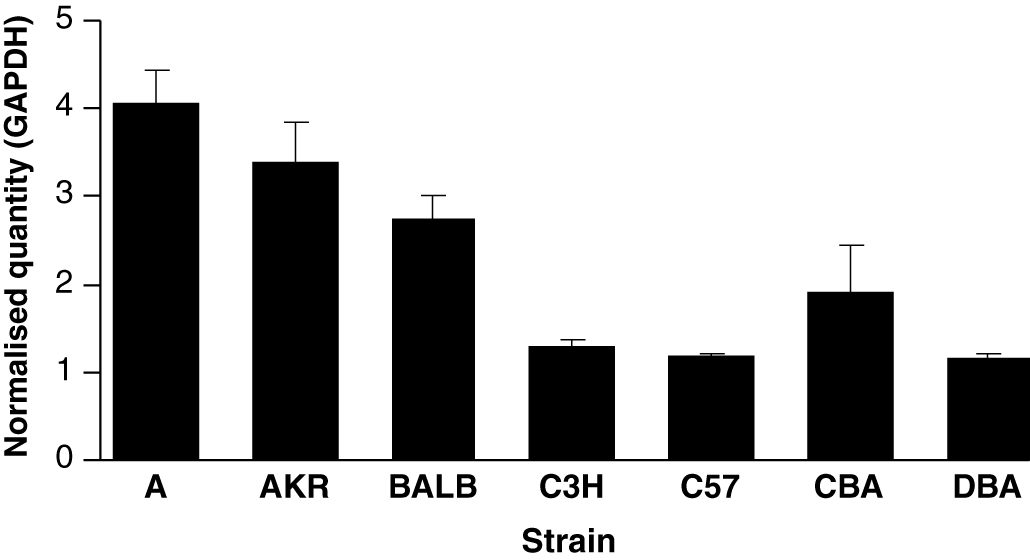

Supplement: Figure S2 — Quantitative RT-PCR of Hectd2 for individual mouse strains. cDNA was prepared from whole brains of uninfected 8 week old male mice or mice at the terminal stages of disease following intracerebral inoculation with Chandler/RML mouse-adapted scrapie prions. All samples were duplexed for Hectd2 and GAPDH fluorogenic probes and run in triplicate with n = 6 for each mouse strain/group. Mean±s.e.m. Hectd2 mRNA expression level is expressed in arbitrary units as normalised by the quantity of GAPDH (y-axis). All mouse strains carry the Prnpa allele. Published incubation times with Chandler/RML are: AKR 123±4; BALB/c 124±11; C3H 132±4; DBA/2 134±3, C57BL/6 137±0 or 143±4; CBA 140±10 [20]–[22]. Incubation times with RML prions are unknown for mouse strains A and LP. (0.02 MB DOC) [file pgen.1000383.s002.doc]
