# Supplementary material for: HECTD2 Is Associated with Susceptibility to Mouse and Human Prion Disease
Source: PLoS Genet. 2009 Feb 13;5(2):e1000383. doi: 10.1371/journal.pgen.1000383 (PMC2633041; doi:10.1371/journal.pgen.1000383)
Supplement: Table S1 — Linkage analysis for microsatellite markers Mmu19. (0.03 MB DOC) [file pgen.1000383.s003.doc]

**Table S1**

**Linkage analysis for microsatellite markers Mmu19**

| **Interval** | **Markers** | **-logP** |
| --- | --- | --- |
| 1 | *D19Mit86-D19Mit99* | 2.60 |
| 2 | *D19Mit99-D19Mit132* | 2.99 |
| 3 | *D19Mit132-D19Mit46* | 2.88 |
| 4 | *D19Mit46-D19Mit63* | 4.92 |
| 5 | *D19Mit63-D19Mit65* | 5.88 |
| 6 | *D19Mit65-D19Mit119* | 3.80 |
| 7 | *D19Mit119-D19Mit90* | 0.66 |
| 8 | *D19Mit90-D19Mit112* | 0.98 |
