# Supplementary material for: HECTD2 Is Associated with Susceptibility to Mouse and Human Prion Disease
Source: PLoS Genet. 2009 Feb 13;5(2):e1000383. doi: 10.1371/journal.pgen.1000383 (PMC2633041; doi:10.1371/journal.pgen.1000383)
Supplement: Table S2 — Trait estimates for HS parental strains D19Mit63-D19Mit65. (0.03 MB DOC) [file pgen.1000383.s004.doc]

**Table S2**

***Trait estimates for HS parental strains D19Mit63-D19Mit65***

| **Strain** | **Effect + se** |
| --- | --- |
| A/J | 131.1 + 35.4 |
| AKR/J | 472.4 + 184.3 |
| BALB/cJ | 131.1 + 35.4 |
| C3H/HeJ | -96.6 + 24.2 |
| C57BL6/J | -113.6 + 172.8 |
| CBA/J | -96.6 + 24.2 |
| DBA/2J | -96.6 + 24.2 |
| LP/J | -96.6 + 24.2 |

se = standard error
