# Supplementary material for: HECTD2 Is Associated with Susceptibility to Mouse and Human Prion Disease
Source: PLoS Genet. 2009 Feb 13;5(2):e1000383. doi: 10.1371/journal.pgen.1000383 (PMC2633041; doi:10.1371/journal.pgen.1000383)
Supplement: Table S3 — Sequenced RefSeq genes from interval D19Mit63-D19Mit65. (0.03 MB DOC) [file pgen.1000383.s005.doc]

**Table S3**

**Sequenced RefSeq genes from interval *D19Mit63-D19Mit65***

| *Htr7* |
| --- |
| *Rpp30* |
| *Ankrd1* |
| *Pcgf5* |
| *Hectd2* |
| *Ppplr3c* |
| *Cpeb3* |
| *March5* |
| *Ide* |
| *Kif11* |
| *Hhex* |
| *Exoc6* |
| *Cyp26c1* |
| *Cyp26a1* |
| *Fer1l3* |
| *Gpr120* |
| *Rpb4* |
| *Pde6c* |
| *Lgil* |
| *Tmem20* |
| *Plce1* |
| *Tb1d12* |
