# Supplementary material for: HECTD2 Is Associated with Susceptibility to Mouse and Human Prion Disease
Source: PLoS Genet. 2009 Feb 13;5(2):e1000383. doi: 10.1371/journal.pgen.1000383 (PMC2633041; doi:10.1371/journal.pgen.1000383)
Supplement: Table S4 — Analysis of polymorphisms from D19Mit63-D19Mit65. (0.3 MB DOC) [file pgen.1000383.s006.doc]

**Table S4**

| **SNP name** | **A** | **AKR** | **BALB** | **C3H** | **C57** | **CBA** | **DBA** | **LP** | **-log P** |
| --- | --- | --- | --- | --- | --- | --- | --- | --- | --- |
| ANKRDIN6 | T | C | T | T | T | T | T | T | 1.97 |
| RPPIN1 | C | G | C | C | C | C | C | C | 1.97 |
| RPPIN3 | T | G | T | T | T | T | T | T | 1.97 |
| RPPIN10 | G | A | G | G | G | G | G | G | 1.97 |
| RPP3UTR | C | T | C | C | C | C | C | C | 1.97 |
| PCGFIN7 | G | G | G | A | A | A | A | A | 6.12 |
| PCGF3U | T | T | T | A | A | A | A | A | 6.12 |
| HECTP1 | G | G | G | A | A | A | A | A | 6.12 |
| HECTP2 | G9 | G9 | G9 | G3 | G3 | G3 | G3 | G3 | 6.12 |
| HECTP3 | T | T | T | C | C | C | C | C | 6.12 |
| HECTP4 | del | del | del | ccgccg | ccgccg | ccgccg | ccgccg | ccgccg | 6.12 |
| HECTP5 | G | G | G | A | A | A | A | A | 6.12 |
| HECTX1 | A | A | A | G | G | G | G | G | 6.12 |
| HECTIN2 | delG | delG | delG | G | G | G | G | G | 6.12 |
| HECTIN3 | A | A | A | G | G | G | G | G | 6.12 |
| HECTIN4 | gctgtt | gctgtt | gctgtt | ac | ac | ac | ac | ac | 6.12 |
| HECTIN5 | C | C | C | C | T | C | C | C | 4.73 |
| HECTIN6 | A | A | A | C | C | C | C | C | 6.12 |
| HECTIN12 | CTAAA | CTAAA | CTAAA | del | del | del | del | del | 6.12 |
| HECTIN13 | G | G | G | A | A | A | A | A | 6.12 |
| HECTIN19 | C | C | C | T | T | T | T | T | 6.12 |
| HECTIN20 (GGTT)n | 8 | 8 | 8 | 11 | 12 | 11 | 11 | 11 | 6.15 |
| HECT3U1 (TA)n | 10 | 10 | 10 | 6 | 6 | 6 | 6 | 6 | 6.12 |
| HECT3U2 | G | G | G | A | A | A | A | A | 6.12 |
| HECT3U3 | A | A | A | G | G | G | G | G | 6.12 |
| HECT3U4 (GTTTT)n | 6 | 6 | 6 | 5 | 5 | 5 | 5 | 5 | 6.12 |
| HECT3U5 | A | A | A | T | T | T | T | T | 6.12 |
| HECT3U6 | T | T | T | A | T | A | A | A | 6.84 |
| HECT3U7 | C | C | C | T | T | T | T | T | 6.12 |
| CPBIN9 | C | C | C | C | T | C | C | C | 4.73 |
| IDEIN23 | A15 | A15 | A15 | A16 | A16 | A16 | A16 | A15 | 5.79 |
| IDEIN15 | A12 | A12 | A12 | A12 | A11 | A12 | A12 | A12 | 4.73 |
| KIFX17 (L755R) | G | G | G | G | T | G | G | G | 4.73 |
| HHEX2 | G | G | G | G | A | G | G | G | 4.73 |
| HHEIN2A | A | A | A | A | G | A | A | A | 4.73 |
| HHEIN2B | T | T | T | T | C | T | T | T | 4.73 |
| HHEX4 | T | T | T | T | A | T | T | A | 0.90 |
| EXOCIN7 | C | C | C | C | T | C | C | C | 4.73 |
| EXOCIN9A | A | A | A | A | G | A | A | A | 4.73 |
| EXOCIN9B | TT | TT | TT | TT | CG | TT | TT | TT | 4.73 |
| EXOCIN9C | G | G | G | G | A | G | G | G | 4.73 |
| EXOC10 | A | A | A | A | G | A | A | A | 4.73 |
| EXOCIN10A | A | A | A | A | G | A | A | A | 4.73 |
| EXOCIN10B | G | G | G | G | T | G | G | G | 4.73 |
| EXOCIN10C | C | C | C | C | A | C | C | C | 4.73 |
| EXOCIN11A | C | C | C | C | T | C | C | C | 4.73 |
| EXOCIN11B | G | G | G | G | A | G | G | G | 4.73 |
| **SNP name** | **A** | **AKR** | **BALB** | **C3H** | **C57** | **CBA** | **DBA** | **LP** | **-log P** |
| EXOCIN12 | G | G | G | G | C | G | G | G | 4.73 |
| EXOCIN14A | G | G | G | G | C | G | G | G | 4.73 |
| EXOCIN14B | ttgac | ttgac | ttgac | ttgac | atgat | ttgac | ttgac | ttgac | 4.73 |
| EXOC15 | C | C | C | C | G | C | C | C | 4.73 |
| EXOCIN15 | A | A | A | A | G | A | A | A | 4.73 |
| EXOCIN16 | T | T | T | T | C | T | T | T | 4.73 |
| EXOCIN18A | A | A | A | A | T | A | A | A | 4.73 |
| EXOCIN18B | C | C | C | C | T | C | C | C | 4.73 |
| EXOCIN18C | G | G | G | G | C | G | G | G | 4.73 |
| EXOC20 | T | T | T | T | C | T | T | T | 4.73 |
| EXOCIN20A | G | G | G | G | A | G | G | G | 4.73 |
| EXOCIN20B | T | T | T | C | T | C | C | T | 6.74 |
| EXOCIN20C | A | A | A | G | G | G | G | A | 5.79 |
| EXOCIN20D | T | T | T | T | C | T | T | T | 4.73 |
| EXOCIN21 | C | C | C | C | T | C | C | C | 4.73 |
| EXOCIN22 | T | T | T | C | C | C | C | T | 5.79 |
| EXOC23A | T | T | T | G | T | G | G | T | 6.74 |
| EXOC23B | T | T | T | A | A | A | A | T | 5.79 |
| EXOC23C | delA | delA | delA | A | A | A | A | delA | 5.79 |
| EXOCIN23A | delA | delA | delA | A | A | A | A | delA | 5.79 |
| EXOCIN23B | CCT | CCT | CCT | del | CCT | del | del | CCT | 6.74 |
| CYP26CX1T18A | A | A | A | G | A | G | G | A | 6.74 |
| CYP26CX2.1 | A | A | A | C | A | C | C | A | 6.74 |
| CYP26CX2.2 | T | T | C | C | T | C | C | T | 4.25 |
| CYP26CX3.1 | C | C | C | T | C | T | T | C | 6.74 |
| CYP26CX3.2 | A | A | A | G | A | G | G | A | 6.74 |
| CYP26CX4Q256R | A | A | A | G | A | G | G | A | 6.74 |
| CYP26CX5 | G | G | G | A | A | A | A | G | 5.79 |
| CYP26AXIN2 | G10 | G11 | G10 | G9 | G10 | G9 | G9 | G10 | 6.00 |
| CYP26A (G202D) | G | G | G | A | G | A | A | G | 6.74 |
| CYP26AXIN6 | T15 | T15 | T15 | T14 | T15 | T14 | T14 | T14 | 6.84 |
| FER5UA | T | T | T | T | C | T | T | T | 4.73 |
| FER5UB | delC | delC | delC | delC | C | delC | delC | delC | 4.73 |
| FER5UC | C | C | C | C | T | C | C | C | 4.73 |
| FER5UD | T | T | T | T | G | T | T | T | 4.73 |
| FER5UE | CTC | CTC | CTC | CTC | delCTC | CTC | CTC | CTC | 4.73 |
| FERX1 | A | A | A | A | G | A | A | A | 4.73 |
| FERIN2A | C | C | C | C | T | C | C | C | 4.73 |
| FERIN2B | C | C | C | C | T | C | C | C | 4.73 |
| FERIN2C | G | G | G | G | A | G | G | G | 4.73 |
| FERIN2D | AT | AT | AT | AT | TTTT | AT | AT | AT | 4.73 |
| FERX3A | T | T | T | T | C | T | T | T | 4.73 |
| FER(L65M) | A | A | A | A | C | A | A | A | 4.73 |
| FERIN3A | G | G | G | G | A | G | G | G | 4.73 |
| FERIN3B | CCAT | CCAT | CCAT | CCAT | TCA | CCAT | CCAT | CCAT | 4.73 |
| FERIN3C | C | C | C | C | A | C | C | C | 4.73 |
| FERIN3D | T | T | T | T | C | T | T | T | 4.73 |
| FERIN3E | AT | AT | AT | AT | GC | AT | AT | AT | 4.73 |
| FER (I92T) | C | C | C | C | T | C | C | C | 4.73 |
| **SNP name** | **A** | **AKR** | **BALB** | **C3H** | **C57** | **CBA** | **DBA** | **LP** | **-log P** |
| FERIN4 | A | A | A | A | C | A | A | A | 4.73 |
| FERX7 | T | T | T | T | C | T | T | T | 4.73 |
| FERIN10 | A10 | A10 | A10 | A10 | A12 | A10 | A10 | A10 | 4.73 |
| FERIN16A | T5 | T5 | T5 | T5 | T3 | T5 | T5 | T5 | 4.73 |
| FERIN16B | GT15 | GT15 | GT15 | GT15 | GT18 | GT15 | GT15 | GT15 | 4.73 |
| FERIN20 | A | A | A | A | C | A | A | A | 4.73 |
| FERIN37 | T | T | T | T | C | T | T | T | 4.73 |
| FER3U | T | T | T | T | C | T | T | T | 4.73 |
| GPRXIN2 | A | A | A | A | G | A | A | A | 4.73 |
| GPRX3A | C | C | C | C | T | C | C | C | 4.73 |
| GPRX3B | T | T | T | T | C | T | T | T | 4.73 |
| RBPIN3A | G | G | G | G | A | G | G | G | 4.73 |
| RBPIN3B | G | G | G | G | A | G | G | G | 4.73 |
| RBPX5A | A | A | A | A | G | A | A | A | 4.73 |
| RBPX5B | C | C | C | C | T | C | C | C | 4.73 |
| RBP3UTR | C | C | C | C | T | C | C | C | 4.73 |
| RBPIN6A | GTdel | GTdel | GTdel | GTdel | Gtins | GTdel | GTdel | GTdel | 4.73 |
| RBPIN6B | CT | CT | CT | CT | del | CT | CT | CT | 4.73 |
| RBPIN6C | A | A | A | A | G | A | A | A | 4.73 |
| PDEXP1 | A | A | A | A | G | A | A | A | 4.73 |
| PDEX1 | G | G | G | G | A | G | G | G | 4.73 |
| PDEIN1A | T | T | T | T | G | T | T | T | 4.73 |
| PDEIN1B | G | G | G | G | A | G | G | G | 4.73 |
| PDEIN1C (GTTTT)n | 2 | 2 | 2 | 2 | 4 | 2 | 2 | 2 | 4.73 |
| PDEX2A | G | G | G | G | A | G | G | G | 4.73 |
| PDEX2B | T | T | T | T | C | T | T | T | 4.73 |
| PDEX2C | G | G | G | G | A | G | G | G | 4.73 |
| PDEIN2 | G | G | G | G | T | G | G | G | 4.73 |
| PDEIN3A | G | G | G | G | A | G | G | G | 4.73 |
| PDEIN3D (GAA)n | 27 | 29 | 27 | 28 | 31 | 28 | 28 | 28 | 5.58 |
| PDEIN3C (AGG)n | 9 | 9 | 9 | 9 | 7 | 9 | 9 | 9 | 4.73 |
| PDEIN3B | G | G | G | G | A | G | G | G | 4.73 |
| PDEIN4A | 8 | 8 | 8 | 8 | 9 | 8 | 8 | 8 | 4.73 |
| PDEIN5A | G | G | G | G | A | G | G | G | 4.73 |
| PDEIN5B | delTC | delTC | delTC | delTC | TC | delTC | delTC | delTC | 4.73 |
| PDEIN7A | T | T | T | T | G | T | T | T | 4.73 |
| PDEIN7B | C | C | C | C | T | C | C | C | 4.73 |
| PDEIN7C | C | C | C | C | T | C | C | C | 4.73 |
| PDEIN7D | C | C | C | C | A | C | C | C | 4.73 |
| PDEIN9A | A | A | A | A | T | A | A | A | 4.73 |
| PDEIN11A | A | A | A | A | G | A | A | A | 4.73 |
| PDEIN12A | T | T | T | T | G | T | T | T | 4.73 |
| PDEIN14A | tgacctgt | tgacctgt | tgacctgt | tgacctgt | del | tgacctgt | tgacctgt | tgacctgt | 4.73 |
| LGIXP1A | T | T | T | T | C | T | T | T | 4.73 |
| LGIXP1A | A | A | A | A | G | A | A | A | 4.73 |
| LGIXIN1 | CA7 | CA7 | CA7 | CA8 | CA7 | CA8 | CA8 | CA7 | 6.74 |
| LGIXIN4 (GTT)n | 8 | 8 | 8 | 12 | 7 | 12 | 12 | 8 | 6.17 |
| LGIX8B | C | C | C | C | A | C | C | C | 4.73 |
| LGIX8C | A | A | A | A | T | A | A | A | 4.73 |
| **SNP name** | **A** | **AKR** | **BALB** | **C3H** | **C57** | **CBA** | **DBA** | **LP** | **-log P** |
| LGIX8D | C | C | C | C | T | C | C | C | 4.73 |
| LGIX8E ggagcg(accacctcctgg) | Ins | Ins | Ins | Ins | no | Ins | Ins | Ins | 4.73 |
| PLCX2A | A | A | A | A | G | A | A | A | 4.73 |
| PLCX2B (S295N) | G | G | G | G | A | G | G | G | 4.73 |
| PLCIN3A | G | G | G | G | T | G | G | G | 4.73 |
| PLCIN3B | T9 | T9 | T9 | T9 | T8 | T9 | T9 | T9 | 4.73 |
| PLCIN5 (TAAA)n | 6 | 6 | 6 | 6 | 5 | 6 | 6 | 6 | 4.73 |
| PLCIN5 (CAAA)n | 5 | 5 | 5 | 3 | 5 | 3 | 3 | 5 | 6.74 |
| PLCX6 | T | T | T | C | T | C | C | T | 6.74 |
| PLCIN6A | G | G | G | A | G | A | A | G | 6.74 |
| PLCIN6B | A | A | A | G | A | G | G | A | 6.74 |
| PLCX7 | T | T | T | C | T | C | C | T | 6.74 |
| PLCIN7A | A | A | A | G | A | G | G | A | 6.74 |
| PLCIN7B | T | T | T | C | T | C | C | T | 6.74 |
| PLCIN7C | T | T | T | C | T | C | C | T | 6.74 |
| PLCIN7D | CT | CT | CT | TC | CT | TC | TC | CT | 6.74 |
| PLCIN7E | delCA | delCA | delCA | CA | delCA | CA | CA | delCA | 6.74 |
| PLCIN7F | G | G | G | A | G | A | A | G | 6.74 |
| PLCIN8A | A | A | A | G | G | G | G | A | 5.79 |
| PLCIN8B | C | C | C | C | T | C | C | C | 4.73 |
| PLCIN8C | T | T | T | C | T | C | C | T | 6.74 |
| PLCX9 | C | C | C | C | T | C | C | C | 4.73 |
| PLCIN9A cctgtggcctac | yes | yes | yes | yes | del | yes | yes | yes | 4.73 |
| PLCIN9B | C | C | C | C | T | C | C | C | 4.73 |
| PLCIN16 | 7 | 7 | 7 | 6 | 6 | 6 | 6 | 7 | 5.79 |
| PLCIN18A | A | A | A | G | A | G | G | A | 6.74 |
| PLCIN18B | G | G | G | G | A | G | G | G | 4.73 |
| PLCIN18C | A | A | A | A | G | A | A | A | 4.73 |
| PLCIN21 | G | G | G | G | A | G | G | G | 4.73 |
| PLCIN25 | A | A | A | A | T | A | A | A | 4.73 |
| TBC3UA | T6 | T6 | T6 | T6 | T8 | T6 | 6 | T6 | 4.73 |
| TBC3UB | A10 | A10 | A10 | A10 | A8 | A10 | A10 | A10 | 4.73 |

**Analysis of polymorphisms from *D19Mit63-D19Mit65***

Abbreviations: IN=intron, X=exon, P=promoter, 5U=5’UTR, 3U=3’UTR, del=deletion, Ins=insert.

ANKRD=*Ankrd1*, RPP=*Rpp30*, PCGF=*Pcgf5*, HECT=*Hectd2*, CPB=*Cpeb3*, IDE=*Ide*, KIF=*Kif11*, HHE=*Hhex*, EXOC=*Exoc6*, CYP26C=*Cyp26c1*, CYP26A=*Cyp26a1*, FER=*Fer1l3*, GPR=*Gpr120*, RBP=*Rbp4*, PDE=*Pde6c*, LGI=*Lgil*, TMEM=*Tmem20*, PLC=*Plce*1, TBC=*Tb1d12*

-logP values are calculated by HAPPY(<http://www.well.ox.ac.uk/happy> )
