# Supplementary material for: HECTD2 Is Associated with Susceptibility to Mouse and Human Prion Disease
Source: PLoS Genet. 2009 Feb 13;5(2):e1000383. doi: 10.1371/journal.pgen.1000383 (PMC2633041; doi:10.1371/journal.pgen.1000383)
Supplement: Table S5 — Primer and probe sequences for mouse polymorphism genotyping. (0.04 MB DOC) [file pgen.1000383.s007.doc]

**Table S5**

Primer and probe sequences for mouse polymorphism genotyping

| **Gene** | **Polymorphism** | **Method** | **Primers and probes** |
| --- | --- | --- | --- |
| *Hectd2* | Promoter (G)n | MegaBACE  (176/182bp) | ACAGCCCGCGACCAACATGG |
|  |  |  | Fam - GGGCTGGCCGCCAGGCTC |
|  | Intron 3 A/G | AD | atctgcatgtcagggctatgc |
|  |  |  | atttataccttcaacttgagatatcttgtctt |
|  |  |  | Vic - agacaaaagt**g**aagacac |
|  |  |  | Fam - catattagacaaaagt**a**aagac |
|  | 3’UTR A/T | AD | CCTCATTTTAAAAGTAAAAACTACTACTGTTACAT |
|  |  |  | GCAATGCTTTAATTTACTATGGCAGATA |
|  |  |  | Fam - CATGAATTTTCT**A**GCTGATT |
|  |  |  | Vic - CATGAATTTTCT**T**GCTGATT |
|  | Exon 1 T18A | AD | TGAGCTGTTTGTCCGTGCTG |
|  |  |  | GCGGAGGGTCCAGAGTTGTT |
|  |  |  | Fam - CCGGCACC**A**CTC |
|  |  |  | Vic - CGGCACC**G**CTCT |
| *Cyp26a1* | Exon 3 G202D | AD | CTGGTCTACCCCGAGGTGAAG |
|  |  |  | AGAGAAGAGATTGCGGGTCATC |
|  |  |  | Vic – CCG**C**CCGCTGGA |
|  |  |  | Fam - CCG**T**CCGCTGGAC |
| *Plce1* | Exon 6 T/C | AD | GTTCTAAAGATGTGGCAGTTCATGG |
|  |  |  | GTGCAAGGCTCGGGTCACTA |
|  |  |  | Fam - CAGTCTGACAT**T**GAGAC |
|  |  |  | Vic - AGTCTGACAT**C**GAGACC |

AD – Allele discrimination using a Real Time PCR machine (Applied Biosystems). All probes were MGB labelled probes from Applied Biosystems. Bases in bold represent the SNP.
