# Supplementary material for: HECTD2 Is Associated with Susceptibility to Mouse and Human Prion Disease
Source: PLoS Genet. 2009 Feb 13;5(2):e1000383. doi: 10.1371/journal.pgen.1000383 (PMC2633041; doi:10.1371/journal.pgen.1000383)
Supplement: Table S6 — Genotyping results for HECTD2 tagging SNPs. (0.04 MB DOC) [file pgen.1000383.s008.doc]

**Table S6**

**Genotyping results for HECTD2 tagging SNPs**

| **Marker** | **Location** | **vCJD** | **UK controls** | **P-value** | **Kuru** | **Multi-exposure**  **Unaffected** | **P-value** |
| --- | --- | --- | --- | --- | --- | --- | --- |
| rs6583769 | 93135420 | 11.1 (226) | 6.0 (1226) | 0.0059 | 21.2 (288) | 31.3 (230) | 0.0088 |
| rs12247672 | 93153909 | 8.1 (234) | 3.8 (1224) | 0.0039 | 12.9 (286) | 14.5 (228) | 0.6138 |
| **rs12249854** | **93176242** | **8.1 (234)** | **3.9 (1202)** | **0.0049** | **20.0 (290)** | **31.3 (230)** | **0.0031** |
| rs11186574 | 93207581 | 5.6 (232) | 2.9 (1228) | 0.0382 | 21.3 (282) | 31.6 (228) | 0.0083 |
| rs11186575 | 93210084 | 6.4 (236) | 3.0 (1214) | 0.0097 | 20.3 (290) | 31.9 (226) | 0.0029 |
| rs4376822 | 93230146 | 8.1 (234) | 3.5 (1224) | 0.0014 | 21.2 (288) | 31.3 (230) | 0.0088 |
| rs1329652 | 93236127 | 7.8 (230) | 4.0 (1216) | 0.0120 | 20.1 (288) | 31.3 (230) | 0.0036 |
| rs11186597 | 93264351 | 7.9 (228) | 4.1 (1234) | 0.0114 | 25.7 (292) | 35.1 (228) | 0.0200 |

For vCJD, UK controls, Kuru and multi-exposure unaffected groups, the minor allele frequency is shown and the numbers in parentheses refer to total alleles genotyped

Human chromosome 10 location (bp) from NCBI build 36.1
